# Open-label phase II study evaluating safety and efficacy of the non-steroidal farnesoid X receptor agonist PX-104 in non-alcoholic fatty liver disease

**DOI:** 10.1007/s00508-020-01735-5

**Published:** 2020-09-15

**Authors:** Stefan Traussnigg, Emina Halilbasic, Harald Hofer, Petra Munda, Tatjana Stojakovic, Günter Fauler, Karl Kashofer, Martin Krssak, Michael Wolzt, Michael Trauner

**Affiliations:** 1 Division of Gastroenterology and Hepatology, Department of Internal Medicine III, Medical University of Vienna, Waehringer Guertel 18–20, 1090 Vienna, Austria; 2 Clinical Institute of Medical and Chemical Laboratory Diagnostics, Medical University of Graz, Graz, Austria; 3 Institute of Pathology, Medical University of Graz, Graz, Austria; 4 High-Field MR Center, Department of Biomedical Imaging and Image-guided Therapy, Medical University of Vienna, Vienna, Austria; 5 Department of Clinical Pharmacology, Medical University of Vienna, Vienna, Austria

**Keywords:** NAFLD, Fatty liver, Therapy, FXR, Insulin resistance

## Abstract

**Background:**

The PX-104 is an oral non-steroidal agonist for the farnesoid X receptor (FXR), a key regulator of bile acid (BA), glucose and lipid homeostasis.

**Aims and methods:**

This single center, proof of concept study evaluated the efficacy, safety and tolerability of PX-104 in non-diabetic NAFLD patients. 12 individuals were treated daily with 5 mg of PX-104 orally for 4 weeks. Serum liver enzymes, insulin sensitivity by clamp like index (CLIX) and hepatic fat by proton ^1^H‑MRS, MRI-PDFF and CAP were assessed. Hepatic energy metabolism and Kupffer cell function were evaluated by phosphorus ^31^P‑MRS and superparamagnetic iron oxide MRI (SPIO-MRI). Other readouts included serum lipids and markers of BA metabolism/signaling besides fecal microbiome and BA analysis.

**Results:**

A significant decrease in ALT (*p* = 0.027; 1‑tailed) and GGT (*p* = 0.019) was observed, without changes in serum alkaline phosphatase or serum lipids. Insulin sensitivity improved in 92% of patients (*p* = 0.02). However, hepatic steatosis measured by PDFF-MRI, ^1^H‑MRS and CAP besides extended serum lipoprotein and BA profiles did not change. NADPH/γATP ratios at ^31^P‑MRS significantly decreased (*p* = 0.022) possibly reflecting reduced hepatic inflammatory stress, but SPIO-MRI remained unchanged. Reduced preponderance of Coriobacteriaceae (*p* = 0.036) correlated with a relative reduction of total fecal BAs. There were no serious adverse events but short intervals of cardiac arrhythmia recorded in 2 patients led to termination of the study.

**Conclusion:**

The non-steroidal FXR agonist PX-104 improved insulin sensitivity and liver enzymes after 4 weeks of treatment in non-diabetic NAFLD patients. Changes in fecal BAs and gut microbiota deserve more extensive investigations.

**Electronic supplementary material:**

The online version of this article (10.1007/s00508-020-01735-5) contains supplementary material, which is available to authorized users.

## Introduction

Non-alcoholic fatty liver disease (NAFLD) and especially non-alcoholic steatohepatitis (NASH) are associated with a higher risk of progression to liver fibrosis, cirrhosis and hepatocellular carcinoma [1–3]. So far, no drug has been approved for the treatment of NAFLD and NASH [4]. The NASH is characterized by metabolic disturbances [5, 6] combined with hepatic inflammation and apoptosis [7, 8] as well as changes in gut microbiota and bile acid metabolism [9, 10]. An efficacious treatment option that simultaneously targets several pathophysiological aspects is still lacking. Nuclear receptors (NR) such as the farnesoid X receptor (FXR) are important regulators of key mechanisms in hepatic lipid and glucose metabolism as well as bile acid (BA) homeostasis, inflammation, fibrogenesis and gut integrity [11].

Upon activation by endogenous BAs or synthetic agonists FXR initiates transcriptional programs regulating BA, glucose and lipid homeostasis [11, 12]. Beyond these metabolic effects, activated FXR facilitates liver regeneration, exerts anti-inflammatory mechanisms, maintains gut mucosal integrity and improves intestinal antibacterial barrier functions, reduces intrahepatic resistance and thereby portal pressure [13, 14]. The first clinically available FXR agonist obeticholic acid (OCA) improved liver enzymes and insulin sensitivity in patients with NAFLD and type II diabetes [15], with a significant histological improvement of NASH and a reduction of liver fibrosis in the consecutive phase IIb trial [16]. Cilofexor [17], another FXR agonist, showed a significant reduction in hepatic steatosis, liver biochemistry, and serum bile acids in patients with NASH after 24 weeks of treatment. As a common side effect of FXR agonists, pruritus was more common in the treatment (14%) than the placebo (4%) group.

The PX-104 is an orally available, synthetic and non-steroidal high-affinity FXR agonist, which—in contrast to OCA—does not undergo enterohepatic circulation. This phase IIa study was the first clinical study with PX-104 administered to NAFLD patients. The primary aim of this study was to evaluate the safety and tolerability of PX-104 and to assess the impact on liver enzymes and hepatic fat. Furthermore, the comprehensive exploratory design of this study aimed to assess the feasibility of the outlined clinical study protocol for the purpose of obtaining data to design and select meaningful multimodal endpoints for subsequent phase II trials in NAFLD patients.

## Patients and methods

**Fig. 1:**
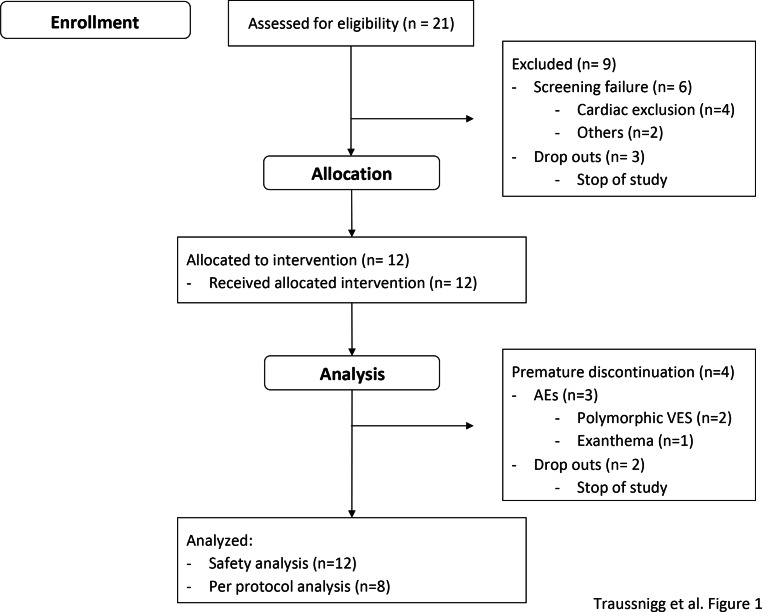
Study enrollment of patients at the Medical University of Vienna (*AE* adverse event, *VES* ventricular extrasystole)

In this study 21 patients were enrolled at the NAFLD outpatient clinic of the Medical University of Vienna (Fig. 1). All patients gave written informed consent to participate in this trial. All versions of the study protocol were approved by the ethics committees of the Medical University Vienna and the Vienna General Hospital Research Ethics Committee (1680/2013). This study is registered at the National Institutes of Health clinical database (clinicaltrials.gov NCT01999101).

### Inclusion and exclusion criteria

Patients enrolled in the study were 18–70 years of age, had a BMI between 25 and 40 and were diagnosed with NAFLD fulfilling all of the following criteria: (1) NAFLD on liver biopsy within 2 years prior to study participation OR liver steatosis on any imaging (ultrasound, MRI, CT), (2) disturbed metabolic homeostasis based on impaired homeostatic model assessment for insulin resistance (HOMA-IR) >1, (3) ALT within or above the upper tertile of the upper limit of normal (ULN; ALT >45U/l for males, >35U/l for females) and/or GGT above ULN (GGT >55U/l for males, >38U/l for females), (4) steatosis >238 db/m at CAP at screening and (5) steatosis >10% equivalent to histology at ^1^H‑MRS at baseline. Exclusion criteria were other causes of liver diseases, history of any structural cardiac disease requiring treatment, any clinical findings in ECG at screening or other confounding conditions (Suppl 1).

### Study design

This open-label phase IIa study was the first clinical study to assess safety and efficacy of PX-104 in NAFLD patients including the evaluation of the outlined procedures in an exploratory sense to plan and design consecutive clinical studies with FXR agonists. Patients were treated with 5 mg of PX-104 orally once daily for 4 weeks according to a previous multiple dose phase I study. The primary objective of this study was to evaluate the safety and tolerability of 5 mg PX-104. Secondary objectives were changes in (1) transaminases, gamma-glutamyl transferase (GGT) and alkaline phosphatase, (2) plasma lipids including serum cholesterol, triglycerides or changes in serum lipoprotein profiles, (3) hepatocellular lipid content and/or changes in liver cell metabolism assessed by ^1^H and ^31^P‑MR spectroscopy, (4) liver stiffness assessed by transient elastography (TE) including controlled attenuation parameter (CAP) validated against MRS, (5) oral glucose tolerance test (oGTT) and/or whole body insulin resistance measured by HOMA index and the clamp-like index (CLIX), (6) phagocytic function of Kupffer cells and possible microcirculatory changes in the liver assessed by supermagnetic iron oxide magnetic resonance imaging (SPIO-MRI) to investigate possible effects on the phagocytic function of Kupffer cells (KCs) and possible microcirculatory changes in the liver, (7) immunological parameters, such as CD3, CD4, CD8 and CD19 assessed by flow cytometry (fluorescence-activated cell sorting, FACS), (8) total BAs and/or BA profiles in serum and stool, including serum 7α-hydroxy-4-cholesten-3-one (C4) and fibroblast growth factor 19 (FGF-19), (9) microbiome analysis as well as (10) intestinal permeability by the sucrose-lactulose-mannitol (SLM) test.

### Methods

Blood samples for hematology, clinical chemistry, coagulation parameters and serology were collected at screening, baseline and weekly until end of treatment and follow-up.

#### Lipoprotein analysis

Lipoproteins were separated using a combined ultracentrifugation precipitation method as described previously [18, 19] (Suppl 2).

#### OGTT, HOMA-IR and CLIX

The OGTT with 75 g of glucose was performed on days −1 and 27 including glucose, insulin and C‑peptide concentrations at 0, 30, 60, 90 and 120 min. These data were used to calculate HOMA-IR and CLIX as described before [20, 21]. In short, CLIX is calculated by using serum creatinine, the AUC of plasma glucose and C‑peptide from OGTT (Suppl 2).

#### ^1^H-MRS and ^31^P-MRS, MRI-PDFF, SPIO

Hepatic fat fraction and hepatic lipid composition were measured using ^1^H‑MRS at 3 T (Trio Tim, Siemens Healthcare, Erlangen, Germany), two dimensional ^31^P‑MR chemical shift imaging (CSI) sequence was assessed at 7 T (Magnetom, Siemens Healthcare). The amplitudes of phosphomonoester [phosphoethanolamine + phosphocholine], phosphodiester [glycerophosphocholine + glycerophosphoethanolamine], uridine diphosphoglucose, nicotine adenine dinucleotide phosphate (NADPH), inorganic phosphate, phosphatidylcholine, α‑adenosine triphosphate and γ‑adenosine triphosphate (ATP), phosphocreatine and total phosphorus (TP) were determined as described before [22–24]. Saturation transfer including chemical exchange rate constant *k* of the Pi to ATP reaction and the unidirectional forward exchange flux (*F*_ATP_) were calculated as published before [25] (Suppl 2).

#### Transient elastography and controlled attenuation parameter (CAP)

Transient elastography (TE) and controlled attenuation parameter (CAP) were performed as described before [26, 27] expressing the results of liver elasticity by liver stiffness (kPa) and success rate (%) and CAP in db/m using the FibroScan® (Echosens, Paris, France) M probe.

#### FACS analysis

Venous drawn EDTA anticoagulated whole blood was stained with monoclonal antibodies using the Simultest-IMK-Plus test according to the manufacturer’s recommendation (Becton Dickinson, San José, CA, USA) (Suppl 2).

#### Bile acid profiles, FGF19 and C4

Bile acids were determined as unconjugated acids and as taurine and glycine conjugates using a high-resolution mass spectrometry method as described previously [28, 29] (Suppl 2). Serum FGF-19 and C4 were measured on baseline and day 28 at several time points.

#### Microbiome

Bacterial DNA from stool samples was extracted with the PowerLyzer PowerSoil DNA Isolation Kit (MO BIO Laboratories, Carlsbad, CA, USA) according to the manufacturer’s instructions. Bacterial 16S rRNA was amplified with the Mastermix 16s Complete PCR Kit (Molzym, Bremen, Germany). The PCR products were subjected to agarose gel electrophoresis and the band of the expected length (330nt) was excised from the gel and purified using the QiaQick (Qiagen, Hilden, Germany) gel extraction system. The DNA concentration of the final PCR product was measured by Picogreen fluorescence (Suppl 2).

#### Sucrose-lactulose-mannitol test

Intestinal permeability was assessed by a triple sugar (sucrose–lactulose–mannitol; SLM) test, as previously validated and described in detail [30] (Suppl 2).

#### Safety

Safety and tolerability assessment were made by monitoring the subjects for adverse and serious adverse events and by interpreting the results of the ECGs (24 h-Holter and 12-lead ECG), various laboratory tests (changes in ALT/AST from baseline) and the subjects’ diaries.

#### Statistical analysis

The main analysis set of interest for evaluation of the primary objective (safety) was the safety population, which included all subjects who received at least one dose of the study drug. The outlined study was conducted as a safety pilot study for a consecutive confirmatory trial. Hence, a formal sample size calculation was not performed. It was anticipated that 12 patients suffering from NAFLD were included in this trial. The sample size was regarded as sufficient to obtain preliminary information about safety and efficacy of the study drug. All efficacy assessments (secondary objectives) aimed to assess the feasibility of the outlined study procedures as well as estimation of the effect size and variability of PX-104 treatment in NAFLD patients in an exploratory way.

For analyses and statistical calculations SPSS® 24.0 (IBM Corp., Chicago, IL, USA) was used. Comparison of continuous variables was calculated using the Student’s t‑test and summarized using the following descriptive statistics: number, mean, standard deviation (SD), minimum, lower quartile (if appropriate), median, upper quartile (if appropriate) and maximum.

## Results

### Patients’ characteristics

A total of 21 patients were enrolled at the Medical University of Vienna as single trial center (Fig. 1), 12 patients were allocated to a therapeutic intervention, 8 patients completed the study and 4 patients were stopped prematurely due to either AEs (*n* = 2) or termination of the study with a median treatment duration of 19.5 days. Demographic data and baseline characteristics are shown in Table 1.

**Table 1:** Baseline characteristics of all patients treated with PX-104

	Safety analysis (*n* = 12)
Age (years)	50 ± 15
Male	8 (66%)
*Comorbidities*	
Hyperlipidemia	7 (58%)
Arterial hypertension	6 (50%)
*Concomitant drug use*	
Antihypertensive	6 (50%)
Anti-lipidemic	0
*Metabolic factors*	
Glucose (mg/dl)	92 ± 11
Insulin (µU/ml)	16 ± 9
Weight (kg)	89 ± 14.4
Body mass index	29.3 ± 3.6
Systolic blood pressure (mm Hg)	127 ± 14
Diastolic blood pressure (mm Hg)	82 ± 6

### Adverse events

Out of 12 patients 11 (92%) experienced at least 1 adverse event (AE). During the study 27 AE were documented. None of these AEs were classified as serious. The AEs were graded as mild (*n* = 26) to moderate (*n* = 1) and 50% of patients experienced at least 1 AE considered as possibly (33%) or probably (17%) related to the study medication. Possibly treatment related AEs >1 patients/AE were diarrhea and headache (*n* = 2). There was no case of itch or pruritus. In 4 patients ventricular extrasystoles (VES) determined by Holter ECG were shown. In two patients the events occurred at the screening visit, classified as screening failures. The relation of study medication to VES was classified as possibly for one patient (polymorphic VES, Lown class 3a), and probably for the second patient (polymorphic VES, Lown class 3a; one ventricular triplet). Subsequent cardiac evaluation by echocardiography revealed no structural cardiac disease in all 5 patients. There was no statistically significant change of QTc from baseline to day 28 recorded from any 12-lead ECG during this trial.

### Liver enzymes and lipid profiles

**Fig. 2:**
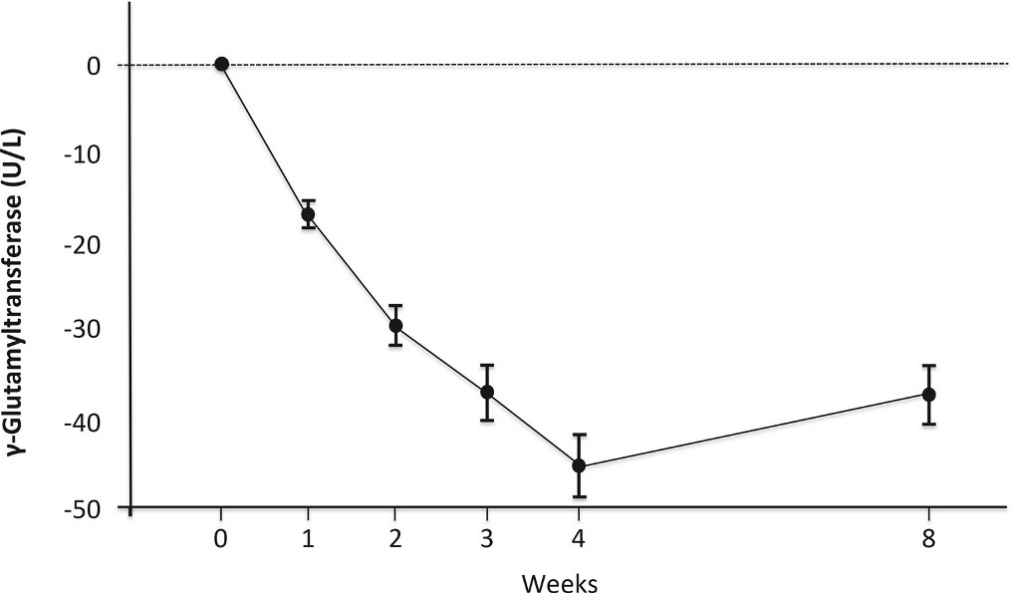
Time course of GGT during treatment and follow-up. Mean values of GGT from baseline to end of treatment (week 4) and follow-up (week 8) during treatment with 5 mg of PX-104 (*n* = 8). Error bars show ± standard deviation

Alanine aminotransferase (ALT) decreased significantly in all but one patient with a mean ALT reduction of 22% (*p* = 0.027, 1‑tailed). All patients had a significant and continuous decrease in gamma-glutamyl transferase (GGT) with a mean reduction of 50% (*p* = 0.0185, 1‑tailed) (Table 2; Fig. 2). Notably, these reductions were followed by a rebound at follow-up (Fig. 2). There was no significant change in aspartate aminotransferase. Importantly, no changes in alkaline phosphatase or bilirubin levels were observed (Table 2). Serum cholesterol including high-density lipoprotein (HDL) and low-density lipoprotein (LDL) besides triglycerides also did not change (Table 2). Additionally, PX-104 had no effect on serum lipid profiles including phospholipid, very low-density lipoprotein or apolipoprotein composition (S Table 1).

**Table 2:** Changes from baseline to end of treatment in body morphometric features, liver enzymes and metabolic parameters

	Mean ± SD		Delta (day 28–day 0)	*P*-values
	Day 0	Day 28		
Weight (kg)	94 ± 14.4	93.8 ± 14.1	−0.3 ± 1.6	0.668
BMI	29.4 ± 4.4	29.3 ± 4.4	−0.1 ± 0.5	0.680
AST (U/L)	34.6 ± 9.1	32.6 ± 4.8	−2.0 ± 7.5	0.240^a^
ALT (U/L)	63.6 ± 25.7	49.8 ± 26	−13.9 ± 17	**0.027**^**a**^
GGT (U/L)	140.4 ± 139.7	70.3 ± 87.2	−70.1 ± 77.1	**0.019**^**a**^
ALP (U/L)	74.5 ± 48.4	76.5 ± 45.8	2 ± 9.4	0.566
Bilirubin (mg/dl)	0.73 ± 0.39	0.67 ± 0.47	−0.06 ± 0.23	0.550
TGL (mg/dl)	144 ± 76	144 ± 61	0.1 ± 32	0.991
TC (mg/dl)	199 ± 46	190 ± 48	−4.9 ± 39.7	0.739
LDL‑C (mg/dl)	119.6 ± 43.4	107 ± 31	−12.5 ± 40.6	0.483
HDL‑C (mg/dl)	42.2 ± 11.7	40 ± 13.3	−2.2 ± 3.5	0.195
HOMA-IR	73.8 ± 46.4	72.2 ± 45.6	−1.6 ± 16.3	0.787

*ALT* Alanine aminotransferase, *AST* Aspartate aminotransferase, *BMI* Body mass index, *GGT* gamma-glutamyl transferase, *HDL-C* High density lipoprotein cholesterol, *HOMAR-IR* Homeostatic Model Assessment for Insulin Resistance, *LDL* Low density lipoprotein cholesterol, *TC* total cholesterol, *TGL* triglycerides

^a^1‑tailed test

### Transient elastography, CAP, PDFF-MRI and MR-Spectroscopy

Hepatic lipid content measured with CAP and ^1^H‑MRS as well as liver stiffness in TE did not change during the treatment period. Nevertheless, hepatic fat fraction decreased in 75% of patients at PDFF-MRI, which was not significant (*p* = 0.126). Furthermore, saturation indices reflecting hepatic saturated, unsaturated and polyunsaturated fatty acids in ^1^H‑MRS remained unchanged; however, the NADPH/γATP ratio at ^31^P‑MRS significantly decreased from a mean of 0.15 ± 0.08 at baseline to 0.10 ± 0.16 at end of treatment (EOT) (*p* = 0.022). Moreover, a significant negative correlation was observed between decreasing levels of GGT and γATP/TP ratios (*p* = 0.033).

### OGTT, HOMA-IR and CLIX

**Fig. 3:**
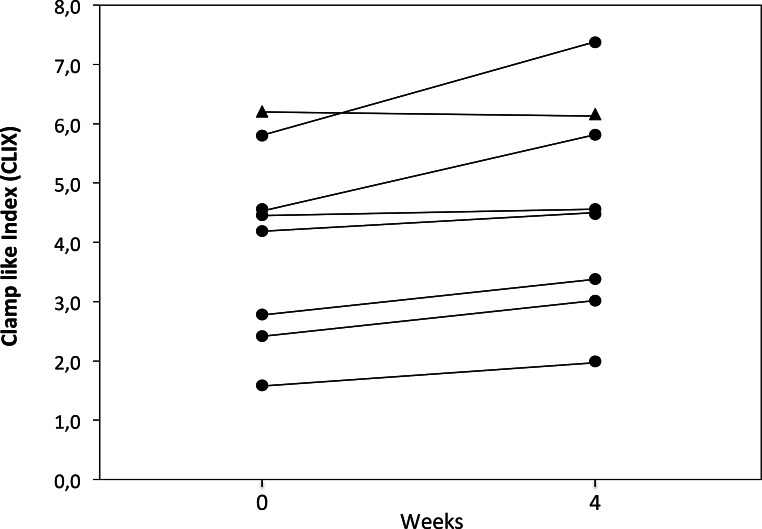
Clamp like index (CLIX) from baseline to end of treatment. Insulin sensitivity assessed by CLIX significantly improved in seven patients (circles) only slightly decreasing in one (triangle)

Although there were no changes in the AUCs of serum glucose, insulin or HOMA-IR, the AUC of C‑peptide measured by OGTT was significantly decreased from baseline to end of treatment (*p* = 0.024). Importantly, clamp-like index as a marker of insulin sensitivity [21] significantly increased (from 3.99 ± 1.62 mg • kg^−1^ • min^−1^ at baseline to 4.59 ± 1.79 mg • kg^−1^ • min^−1^ at EOT; *p* = 0.02; Fig. 3) with a defined threshold for insulin resistance of <5 mg • kg^−1^ • min^−1^.

### SPIO and immunology

Measurements by SPIO-MRI reflecting liver macrophage/inflammatory activity did not show any changes with PX-104 treatment; however, we observed a significant decrease in serum CD3-/HLA-DR+ ratio (mainly B‑lymphocytes) in FACS analysis (15.03 ± 2.42 vs. 12.34 ± 2.69, *p* = 0.027), while other subsets (e.g. CD 4, CD 8, CD 19) remained unchanged (S Table 2). The SPIO-MRI significantly correlated with classical monocytes in serum (r = 0.775; *p* = 0.024), which were increased in 75% of patients (*p* = 0.584) and correlated negatively with intermediate monocytes (r = −0.705; *p* = 0.051) (S Fig. 1).

### Bile acid profiles

No changes in serum total bile acid (BA) levels or serum BA profiles were observed. Nevertheless, total fecal BAs decreased in 88% of patients, which did not reach significance (*p* = 0.151) (Table 3); however, we observed changes at the levels of individual BAs. More specifically, fecal conjugated primary BAs decreased (*p* = 0.051) including reduced levels of cholic acid (CA; *p* = 0.075) and chenodeoxycholic acid (CDCA; *p* = 0.049). Interestingly, we could observe a trend for increasing levels of fecal lithocholic acid (LCA) with a significant rise of tauro-lithocholic acid (TLCA; *p* = 0.049). Additionally, changes in ^1^H‑MRS hepatic fat fraction correlated positively with serum secondary unconjugated BAs (r = 0.882, *p* = 0.004) and negatively with serum secondary conjugated BAs (−0.752, *p* = 0.031) (S Fig. 3). C4 decreased significantly after administration from time point 0 to 6 h from 31.5 ± 10.2 ng/ml to 16.9 ± 12.1 ng/ml at day 0 (*p* = 0.0055, 1‑tailed) and from 21.4 ± 11.4 ng/ml to 12.7 ± 6.0 ng/ml at day 28 (*p* = 0.0315, 1‑tailed). Accordingly, FGF-19 increased significantly from time point 0 h to the time point of the maximum individual increase (between time points 2 and 6 h) 100.6 ± 60.1 pg/ml to 526.7 ± 278.0 pg/ml at day 0 (*p* = 0.00045; 1‑tailed) and from 89.9 ± 33.9 pg/ml to 651.3 ± 214.9 pg/ml at day 28 (*p* = 0.0005; 1‑tailed) whereas neither C4 nor FGF-19 concentrations significantly changed over time between day 0 and 28.

**Table 3:** Changes from baseline to end of treatment in fecal bile acid profiles

	Mean ± SD			
Bile acid (nmol/g)	Day 0	Day 28	Delta (day 28–day 0)	*P*-values
Unconjugated primary BAs	14,991 ± 23,075	5645 ± 8425	−9345 ± 23,352	0.295
CA	8587 ± 12,850	3777 ± 6122	−4809 ± 13,268	0.339
CDCA	6405 ± 10,250	1868 ± 2481	−4536 ± 10,302	0.253
Unconjugated secondary BAs	101,956 ± 95,530	62,217 ± 35,861	−39,738 ± 84,682	0.226
DCA	60,510 ± 54,809	34,570 ± 22,537	−25,940 ± 49,197	0.180
LCA	37,583 ± 45,774	26,183 ± 15,718	−11,400 ± 38,185	0.426
UDCA	3864 ± 5595	1466 ± 3013	−2398 ± 3836	0.120
Primary to secondary ratio	0.81 ± 1.95	0.16 ± 0.27	−0.65 ± 1.70	0.314
Total BAs	118,447 ± 92,312	68,742 ± 40,009	−49,706 ± 87,217	0.151
Conjugated primary BAs	1004.7 ± 774.8	455.2 ± 282.5	−549.5 ± 660.7	*0.051*
GCA	320.7 ± 386.5	151.6 ± 127.2	−169.2 ± 305.1	0.161
TCA	179.6 ± 293.7	56.6 ± 44.5	−123.0 ± 294.1	0.275
*Total conjugated CA*	500.4 ± 458.8	208.15 ± 156.7	−292.2 ± 395.3	0.075
GCDCA	380.6 ± 314.7	180.2 ± 104.6	−200.4 ± 266.0	0.070
TCDCA	123.7 ± 87.8	66.9 ± 32.6	−56.9 ± 82.6	0.093
*Total conjugated CDCA*	504.3 ± 350.3	247 ± 132.3	−257.3 ± 305.8	*0.049*
Conjugated secondary BAs	569.6 ± 437.5	478.8 ± 280.8	−90.8 ± 352.1	0.489
GDCA	405.4 ± 369.2	293.0 ± 188.6	−112.4 ± 288.0	0.306
TDCA	46.9 ± 57.3	63.4 ± 79.0	16.5 ± 29.2	0.153
*Total conjugated DCA*	452.3 ± 386.6	356.4 ± 216.2	−95.9 ± 305.1	0.404
GLCA	33.8 ± 61.5	47.2 ± 42.8	13.4 ± 34.7	0.312
TLCA	8.4 ± 16.7	19.0 ± 26.4	10.6 ± 12.6	*0.049*
*Total conjugated LCA*	42.2 ± 59.9	66.2 ± 51.0	24.0 ± 42.4	0.154
GUDCA	47.4 ± 45.0	25.2 ± 39.3	−22.1 ± 39.6	0.158
TUDCA	27.7 ± 16.5	31.0 ± 35.7	3.3 ± 27.7	0.750
*Total conjugated UDCA*	75.1 ± 56.6	56.2 ± 74.1	18.9 ± 62.6	0.422

*BA* bile acid, *CA* cholic acid, *CDCA* chenodeoxycholic acid, *DCA* deoxycholic acid, *GCA* glyco-cholic acid, *GCDCA* glycol-chenodeoxycholic acid, *GDCA* glycol-deoxycholic acid, *GLCA* glycol-lithocholic acid, *GUDCA* glycol-ursodeoxycholic acid, *LCA* lithocholic acid, *TCA* tauro-cholic acid, *TCDCA* tauro-chenodeoxycholic acid, *TDCA* tauro-deoxycholic acid, *TLCA* tauro-lithocholic acid, *TUDCA* tauro-ursodeoxycholic acid, *UDCA* ursodeoxycholic acid

### Microbiome and SLM test

We observed a significant decrease in *Coriobacteriaceae* in relation to the total bacteria (*p* = 0.036) with an additional trend of decreasing levels of proteobacteria (*p* = 0.071) including *Enterobacteriacecae* (*p* = 0.071) (Table 4). A relative decrease of *Coriobacteriaceae* was associated with a relative reduction of fecal total BAs (r = 0.838, *p* = 0.009), mainly primary (r = 0.744, *p* = 0.034) and secondary unconjugated BAs (r = 0.835, *p* = 0.01) and showed a negative correlation to conjugated to unconjugated serum BA ratio.

**Table 4:** Changes from baseline to end of treatment in gut microbiota

Bacteria^(k)^				Mean change from baseline (relative to the total number of bacteria)	*P*-value
				(95% CI)	
Actinobacteria^(p)^				–	Ns
	Coriobacteriia^(c)^			–	–
		Coriobacteriales^(o)^		–	–
			Coriobacteriacecae^(f)^	−0.0078 (−0.0007 to −0.0150)	*0.036*
Bacteroidetes^(p)^				–	Ns
Firmicutes^(p)^				–	Ns
Proteobacteria^(p)^				−0.0641 (−0.0074 to −0.1356)	0.071
	Gammaproteobacteria^(c)^			−0.0509 (−0.0143 to −0.1162)	0.107
		Enterobacteriales^(o)^		−0.0469 (−0.0052 to −0.0989)	0.071
			Enterobacteriacecae^(f)^	−0.0469 (−0.0052 to −0.0989)	0.071

*k* kingdom, *p* phylum, *c* class, *o* order, *f* family

No change in *Bacteroidetes or Firmicutes* was observed; however, the class of *Bacilli *of the phylum *Firmicutes* correlated with ^1^H‑MRS hepatic fat fraction (r = 0.721, *p* = 0.044) (S Fig. 2), the relative portion of secondary unconjugated BAs (r = 0.760, *p* = 0.029) and total BAs (r = 0.780, *p* = 0.022). Furthermore, the SLM test was unremarkable and did not change over time.

## Discussion

This is the first proof-of-concept phase II trial assessing the safety and efficacy of the non-steroidal FXR agonist PX-104 in patients with NAFLD demonstrating a significant decrease of GGT and ALT levels together with an improvement of insulin sensitivity. Interestingly, these therapeutic effects were accompanied by significant changes in fecal BA profiles and microbiota composition.

A continuous decrease of GGT as an indicator of oxidative stress and strong predictor of overall mortality [31] was observed in all patients under PX-104 treatment, with a subsequent rebound at follow-up. Similar results could be shown for ALT suggesting beneficial effects of PX-104 on liver injury. Notably, we did not find any changes in BMI or lipid profiles. Activation of FXR may lower HDL levels [32] and increase LDL as a consequence of repressed LDL receptor expression [33], which has also been reported in patients treated with steroidal FXR agonists such as obeticholic acid (OCA) [16]. Importantly, in this trial there was no significant change in levels of serum cholesterol, HDL or LDL possibly reflecting important differences between steroidal and non-steroidal FXR ligands besides varying treatment doses, duration and a small sample size.

The CAP, PDFF-MRI and ^1^H‑MRS did not show any significant treatment-associated changes in hepatic lipid content or lipid saturation indices. Although we observed a trend towards a reduction of liver fat, 4 weeks of treatment might have been too short to observe significant changes in steatosis. Likewise, liver stiffness evaluated by transient elastography did not change. Notably, using more sensitive methods, such as ultrahigh-field 7T MRS, the NADH/γATP ratio in ^31^P‑MRS was significantly decreased after treatment with PX-104 with a negative correlation between GGT levels and γATP/TP ratios possibly indicating reduced inflammatory stress and an improved hepatic energy metabolism [34].

Importantly, we observed a significant improvement of insulin sensitivity assessed by CLIX, which highly correlated with clamp glucose infusion rates, the current gold standard to assess insulin sensitivity [21]. Notably, the study population did not include patients with diabetes and perhaps even more pronounced effects might be expected in a NAFLD population with diabetes as observed with OCA [15].

In addition to the metabolic effects on hepatic BA, glucose and lipid homeostasis, FXR ligands may have also profound impact on innate and adaptive immunity [35] making them an interesting class of immunometabolic drugs. To further evaluate possible effects on hepatic inflammation, flow cytometry and SPIO-MRI were performed. Using FACS analysis we found a significant reduction of serum CD3-/HLA-DR+ cells. Interestingly, although the CD3-/HLA-DR+ population describes in the majority B‑lymphocytes it may also contain activated NK lymphocytes, monocytes and/or macrophages, which have all been implicated in the pathogenesis and progression of NALFD/NASH [36].

With an increasing number of CD14 positive Kupffer cells in the liver and growing hepatic necroinflammation, the reduction of liver-to-muscle signal intensity ratio (reduction-%LMR) decreases in SPIO-MRI [37]. In our patients SPIO-MRI signals did not change over time; however, decreasing levels in reduction-%LMR correlated with lower levels of classical serum monocytes and more importantly higher levels of intermediate monocytes. The CD14++CD16+ intermediate monocytes play an important role in chronic liver inflammation recruited from blood and locally differentiated from classical CD14++CD16− monocytes [38] showing possible effects on immune modulation, which is an important trigger of hepatic inflammation [39].

Total fecal BAs declined in all patients but one, including conjugated primary BAs, whereas serum BAs did not change. Interestingly, total fecal BAs are higher in NASH patients than healthy controls including unconjugated CA and CDCA [40]. Recent data implicate an important role of FXR in modulating microbiota also associated with changes in BA profiles [41]. Importantly, PX-104—in contrast to steroidal FXR ligands such as OCA—does not undergo enterohepatic circulation and microbial BA metabolism. Notably, several hours after administration of PX-104, serum concentrations of C4 significantly decreased whereas serum FGF-19 (only measured in 6/8 patients) significantly increased supporting an FXR agonistic effect; however, neither serum levels of C4 nor FGF-19 significantly changed over time, indicating a lacking cumulative effect implying alternative mechanisms, which deserve further studies.

In our patients *Coriobacteriaceae* family significantly decreased over time. *Coriobacteriaceae* might play a role in BA metabolism [42] and was also shown to correlate with intrahepatic fat in mice [43]. In the current study a relative decrease of *Coriobacteriaceae* was significantly associated with a relative reduction of fecal total BAs, mainly primary and secondary unconjugated BAs. Interestingly, only one patient showed an increase of *Coriobacteriaceae*, which was accompanied by the only increase of fecal total BAs in our patients also standing out by a reduced conjugated to unconjugated ratio in fecal BAs. Intestinal and hepatic FXR activation may have differential effects on hepatic fat [44]. Interestingly, the changes of hepatic fat fraction in ^1^H‑MRS correlated positively with total serum BAs and unconjugated secondary serum BAs, but negatively with conjugated secondary serum BAs. Moreover, the class of *Bacilli *of the phylum *Firmicutes* correlated with ^1^H‑MRS hepatic fat fraction and total serum BAs, suggesting potential functional/mechanistic links. Current data show a significant association of improved hepatic fat and a reduction of *Firmicutes* after 6 months of treatment with probiotics [45]. Recently, the gut microbiota including *Firmicutes* were also linked to diet-induced obesity via alterations in BA profiles with altered FXR signaling [41]. Although several alterations in serum and fecal BA profiles as well as fecal microbiota composition could be observed in this study, the role of FXR modulation and associated alterations in microbiota is unclear and deserves further studies.

In this trial, the majority of AEs were mild and tolerability of the study drug was graded as good with no withdrawal of consent. We did not observe any pruritus or a significant increase in serum cholesterol or LDL, common side effects in steroidal FXR agonists [16]. Nevertheless, one patient experienced an exanthema with a non-recurring episode of fever and two patients showed isolated polymorphic premature ventricular contractions with a singular ventricular triplet in one patient without symptoms leading to drop-out in each case due to study protocol. A narrow ECG surveillance was part of the safety protocol as cardiac arrhythmias were reported in phase I for one subject with an unclear relation to the drug application. Abnormalities in Holter-ECG during the treatment phase were rated possibly in one and probably in another subject. According to this grading a relationship between study medication and VES could not be ruled out leading to the termination of the study after 12 patients without replacement of previous drop-outs. Nevertheless, occasionally occurring VES in healthy volunteers are recorded in up to 60% of 24 h Holter-ECGs [46]. Accordingly, recent data on 1273 healthy volunteers from 22 phase I studies showed VES in 43% of subjects [47]. Considering ECG abnormalities in three other patients during screening visits leading to an exclusion from the study and given that data support a strong association between NAFLD and cardiovascular and/or arrhythmic complications [48], these findings may not rule out a relationship with the study medication but should be considered in subsequent phase II studies including 24 h Holter-ECG examinations.

The present study has several limitations with a low number of patients, short treatment duration of 4 weeks, rather low dosage of the study drug and no randomization to placebo controls. Additionally, liver biopsy was not required to diagnose NAFLD owing the pilot character of the study. The strength of this trial was a very comprehensive metabolic, functional imaging and immunological characterization of NAFLD patients testing promising non-invasive parameters to be evaluated in longer phase II trials.

In conclusion, this was the first trial of a non-steroidal FXR agonist in non-diabetic NAFLD patients. The use of PX-104 improved insulin sensitivity and decreased serum GGT and ALT levels after 4 weeks of treatment without increasing serum cholesterol or alkaline phosphatase; however, there was no change in hepatic steatosis measured by ^1^H‑MRS and PDFF-MRI. Furthermore, we observed changes in immunology, BA metabolism and microbiota composition, which was not yet shown for FXR agonists, suggesting that FXR ligands could be interesting immunometabolic drugs in humans. A relationship between PX-104 and cardiac arrhythmia could not be ruled out. Further studies with follow-up compounds (e.g. cilofexor) showing improved FXR efficacy, significant reductions in hepatic steatosis, liver biochemistry and safety profile appears justified. Overall, this multimechanistic proof-of-concept study shows the clinical relevance developing synthetic non-steroidal FXR agonists in the treatment of metabolic liver diseases.

## Caption Electronic Supplementary Material

*S Fig 1. Serum monocytes in correlation to SPIO MRI during treatment. *With decreasing signals in super paramagnetic iron oxide (SPIO) MRI (associated with higher necroinflammation) classical monocytes decreased and C14++CD16+ intermediate monocytes increased.

*S Fig 2. Changes in serum BA profiles and microbiota associated with hepatic fat change. *Patients with increasing levels of hepatic steatosis after treatment with PX-104 show significantly higher levels of total serum BAs also associated with a relative change in the gut microbiota (significantly higher Bacilli).

*S Fig 3. Changes in BA profiles associated with hepatic fat change.* Patients with increasing levels of hepatic steatosis after treatment with PX-104 show significantly higher levels of serum second unconjugated BAs which was inversely correlating to serum second conjugated BAs.

S Table 1. Changes from baseline to end of treatment in serum lipids and lipoproteins.

S Table 2. Changes from baseline to end of treatment in serum immunological markers.

Suppl 1 File. Detailed inclusion and exclusion criteria.

Suppl 2 File. Methods.

## Abbreviations

ALT: Alanine aminotransferase
AST: Aspartate aminotransferase
ATP: Adenosine triphosphate
BA: Bile acid
BMI: Body mass index
C4: 7α-Hydroxy-4-cholesten-3-one
CA: Cholic acid
CAP: Continuous attenuation parameter
CDCA: Chenodeoxycholic acid
CDT: Carbohydrate deficient
CLIX: Clamp-like index
CSI: Chemical shift imaging
ER: Endoplasmic reticulum
FA: Fatty acid
F_ATP_: forward exchange flux
FGF-19: Fibroblast growth factor 19
FXR: Farnesoid X receptor
GGT: gamma-glutamyl transferase
GPC: Glycerophosphocholine
κ: Exchange rate constant
HDL: High density lipoprotein
LCA: Lithocholic acid
LDL: Low density lipoprotein
MRS: Magnetic resonance spectroscopy
MRI-PDFF: Magnetic resonance imaging-proton density fat fraction
MUI: Monounsaturation index
NADPH: Nicotinamide adenine dinucleotide phosphate
NAFLD: Non-alcoholic fatty liver disease
NASH: Non-alcoholic steatohepatitis
OCA: Obeticholic acid
OGTT: Oral glucose tolerance test
PDE: Phosphodiester
PE: Phosphoethanolamine
Pi: Inorganic phosphate
PME: Phosphomonoester
PUFA: Polyunsaturated fatty acid
PUI: Polyunsaturation index
REC: Research ethics committee
SI: Saturation index
SPIO-MRI: Superparamagnetic iron oxide MRI
TLCA: Tauro-LCA
TE: Transient elastography
TP: Total phosphorus
ULN: upper limit of normal
VES: Ventricular extrasystole
VOI: Volume of interest
WHR: Waist-to-hip ratio
