# Supplementary figures and images for: Open-label phase II study evaluating safety and efficacy of the non-steroidal farnesoid X receptor agonist PX-104 in non-alcoholic fatty liver disease
Source: Wien Klin Wochenschr. 2020 Sep 15;133(9):441–51. doi: 10.1007/s00508-020-01735-5 (PMC8116226; doi:10.1007/s00508-020-01735-5)

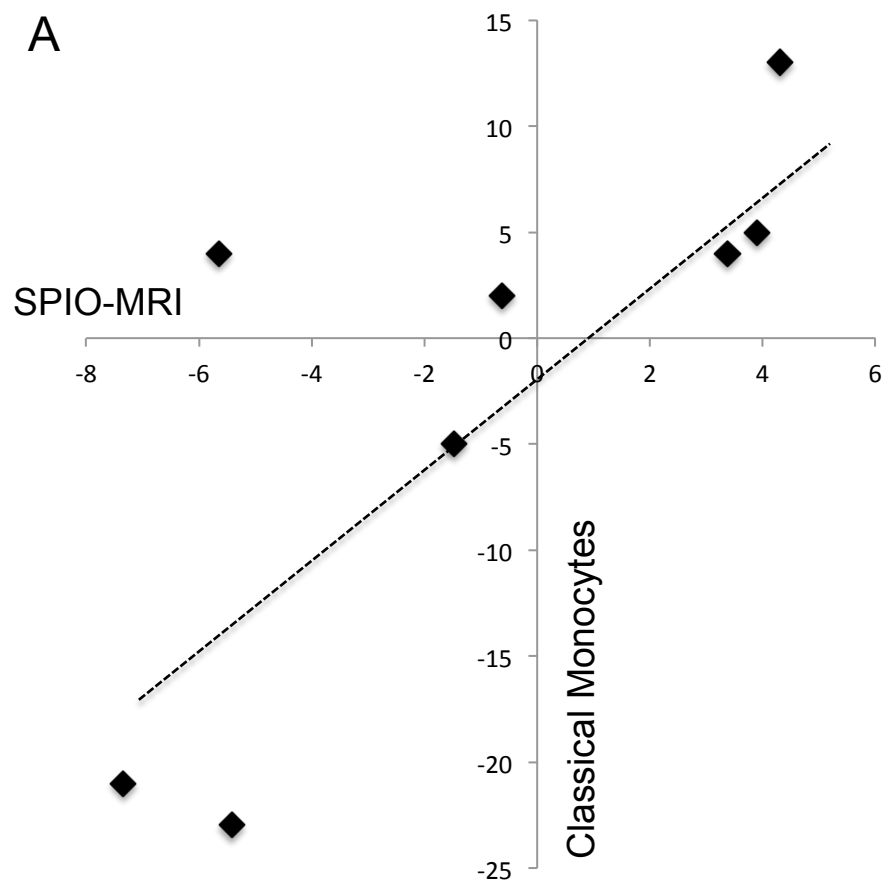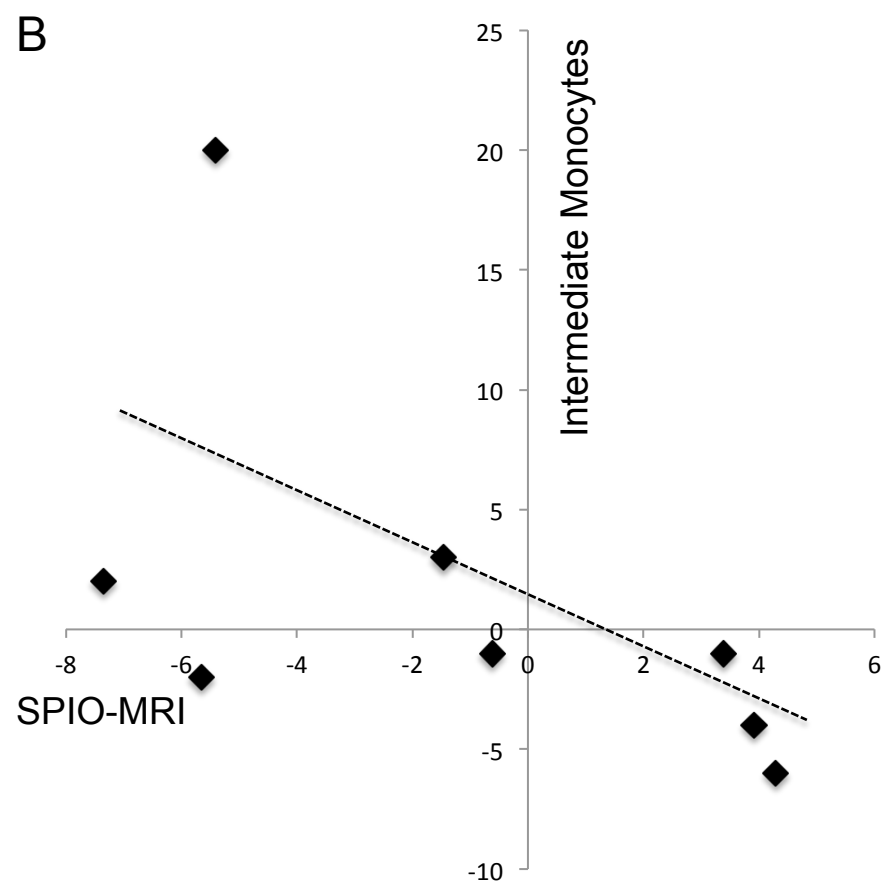

Supplement: Supplementary file 1 — S Fig 1. Serum monocytes in correlation to SPIO MRI during treatment. With decreasing signals in super paramagnetic iron oxide (SPIO) MRI (associated with higher necroinflammation) classical monocytes decreased and C14++CD16+ intermediate monocytes increased. [file 508_2020_1735_MOESM1_ESM.pdf]

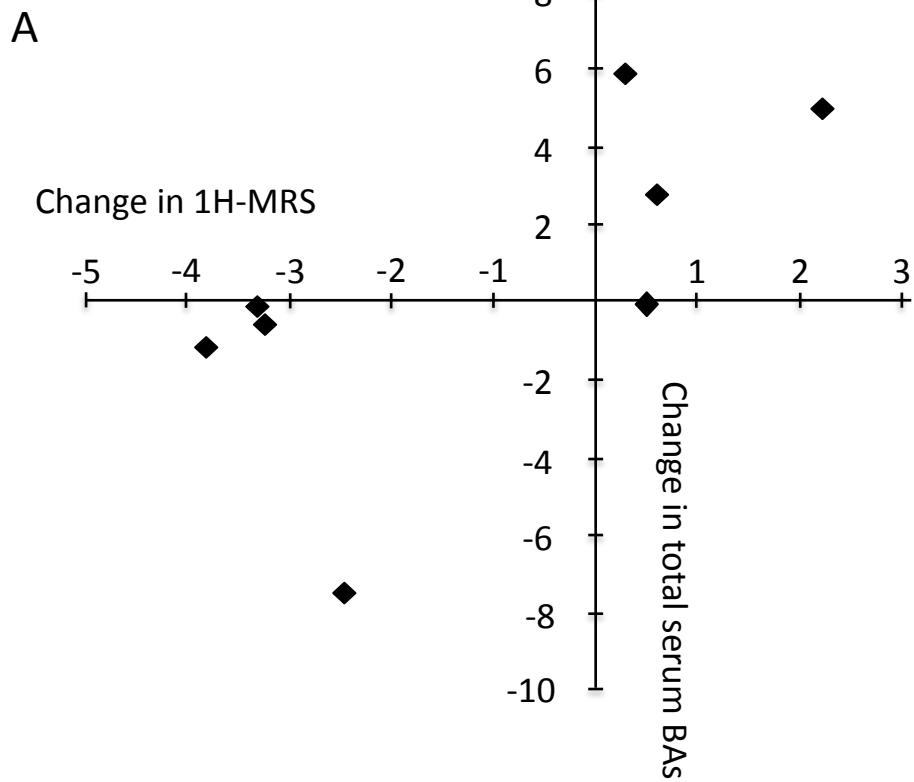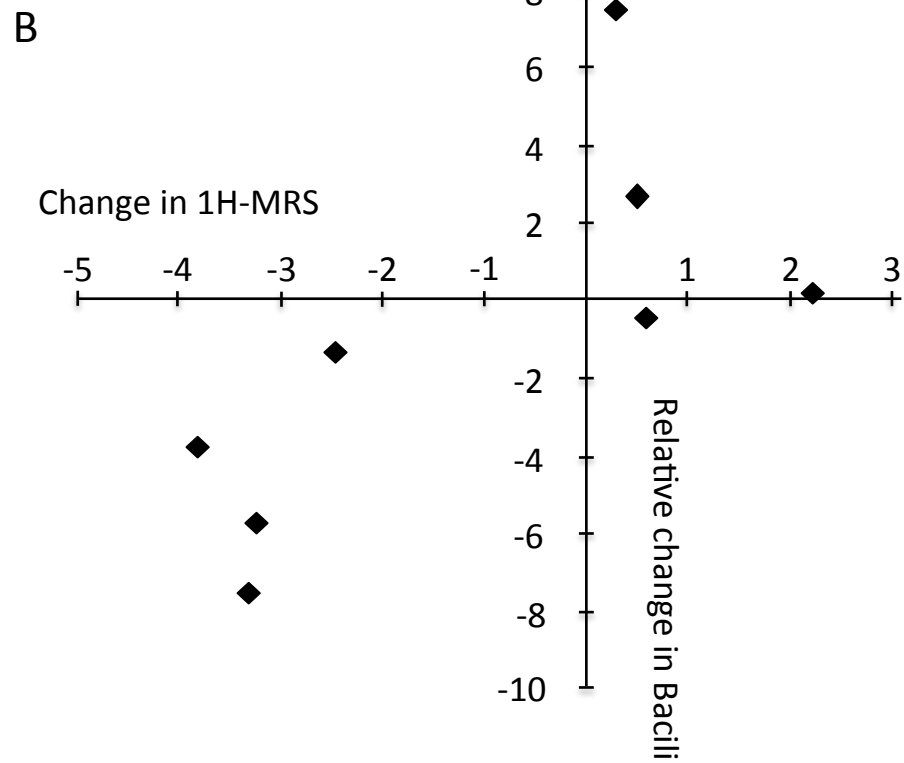

Supplement: Supplementary file 2 — S Fig 2. Changes in serum BA profiles and microbiota associated with hepatic fat change. Patients with increasing levels of hepatic steatosis after treatment with PX-104 show significantly higher levels of total serum BAs also associated with a relative change in the gut microbiota (significantly higher Bacilli). [file 508_2020_1735_MOESM2_ESM.pdf]

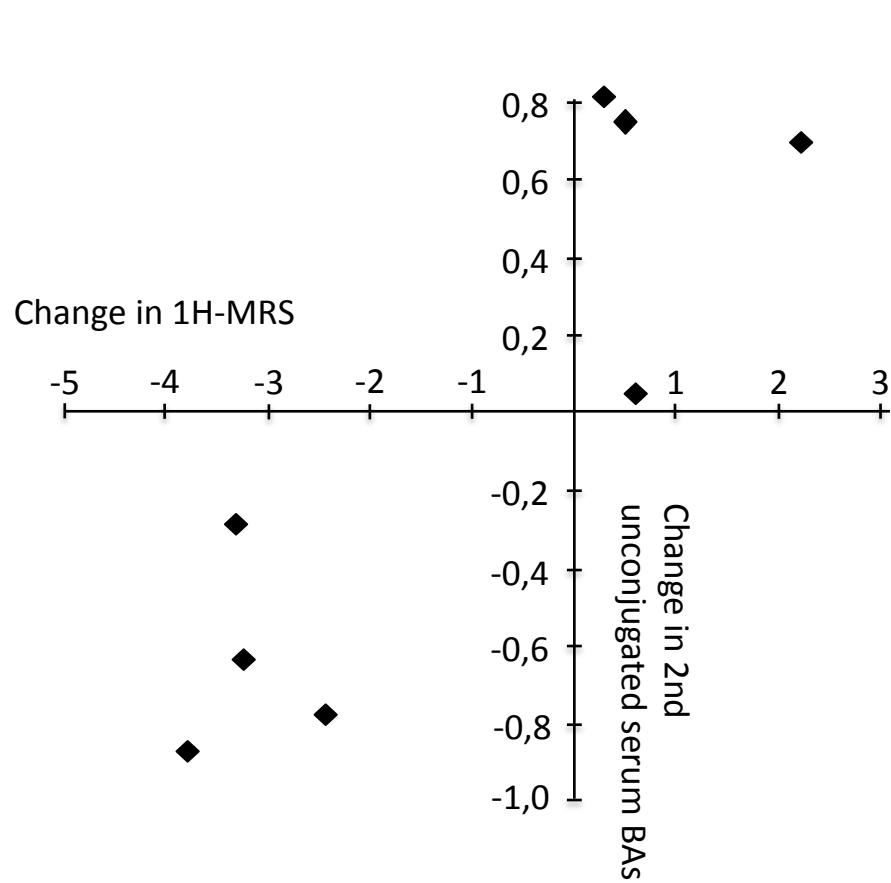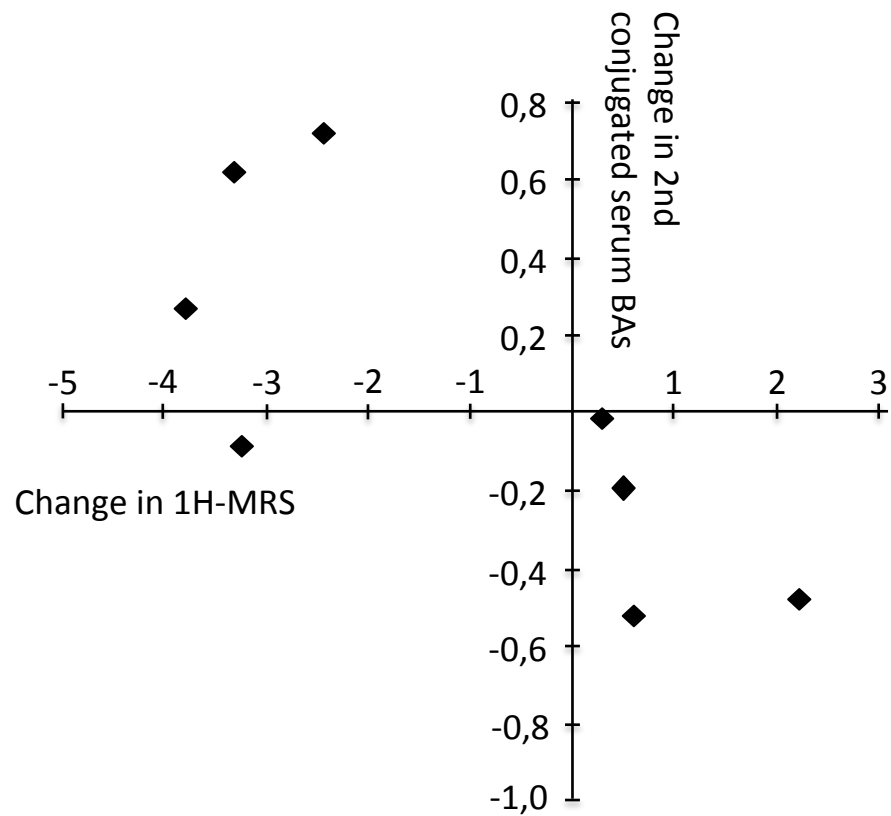

Supplement: Supplementary file 3 — S Fig 3. Changes in BA profiles associated with hepatic fat change. Patients with increasing levels of hepatic steatosis after treatment with PX-104 show significantly higher levels of serum second unconjugated BAs which was inversely correlating to serum second conjugated BAs. [file 508_2020_1735_MOESM3_ESM.pdf]
