# Supplementary material for: Open-label phase II study evaluating safety and efficacy of the non-steroidal farnesoid X receptor agonist PX-104 in non-alcoholic fatty liver disease
Source: Wien Klin Wochenschr. 2020 Sep 15;133(9):441–51. doi: 10.1007/s00508-020-01735-5 (PMC8116226; doi:10.1007/s00508-020-01735-5)
Supplement: Supplementary file 4 — S Table 1. Changes from baseline to end of treatment in serum lipids and lipoproteins. [file 508_2020_1735_MOESM4_ESM.docx]

Supplementary Table 1. Changes from baseline to end of treatment in serum lipids and lipoproteins

| **Serum lipids and lipoproteins** | | **Mean ± SD** | | | | | | |
| --- | --- | --- | --- | --- | --- | --- | --- | --- |
|  |  | **Day 0** | | **Day 28** | | **Delta (d28-d0)** | | **p-value** |
| Serum | Free fatty acids (mmol/l) | 0,53 | ±0,20 | 0,58 | ±0,22 | 0,05 | ±0,20 | 0,486 |
|  | Lipoprotein (a) (mg/dl) | 5,9 | ±11,0 | 6,9 | ±9,7 | 1,0 | ±5,6 | 0,631 |
|  | Apo AI (mg/dl) | 113,1 | ±15,5 | 111,0 | ±14,1 | -2,1 | ±4,9 | 0,258 |
|  | Apo AII (mg/dl) | 42,5 | ±4,0 | 42,1 | ±5,5 | -0,4 | ±2,7 | 0,703 |
|  | Apo B (mg/dl) | 93,1 | ±25,5 | 89,0 | ±23,0 | -4,1 | ±19,2 | 0,563 |
|  | Apo CII (mg/dl) | 4,4 | ±1,8 | 4,4 | ±1,7 | 0 | ±0,9 | 1 |
|  | Apo III (mg/dl) | 12,6 | ±4,7 | 13,3 | ±4,8 | 0,7 | ±3,1 | 5,28 |
|  | Apo E (mg/dl) | 15,5 | ±4,8294 | 16,6 | ±6,2 | 1,2 | ±3,2 | 0,343 |
| VLDL | Cholesterol (mg/dl) | 41 | ±33 | 39 | ±29 | -2 | ±7 | 0,599 |
|  | Triglycerides (mg/dl) | 192 | ±271 | 157 | ±171 | -35 | ±104 | 0,371 |
|  | Apo B (mg/dl) | 12,5 | ±9,1 | 13,5 | ±8,4 | 1,0 | ±3,9 | 0,487 |
| LDL | Cholesterol (mg/dl) | 120 | ±40 | 110 | ±32 | -10 | ±32 | 0,412 |
|  | Triglycerides (mg/dl) | 33 | ±9 | 32 | ±7 | -1 | ±6 | 0,719 |
|  | Apo B (mg/dl) | 80,6 | ±21,8 | 75,5 | ±19,6 | -5,1 | ±18,8 | 0,467 |
| HDL | Cholesterol (mg/dl) | 34 | ±8 | 32 | ±11 | -2 | ±4 | 0,34 |
|  | Triglycerides (mg/dl) | 15 | ±5 | 14 | ±3 | 0 | ±3 | 0,765 |
