# Supplementary material for: Open-label phase II study evaluating safety and efficacy of the non-steroidal farnesoid X receptor agonist PX-104 in non-alcoholic fatty liver disease
Source: Wien Klin Wochenschr. 2020 Sep 15;133(9):441–51. doi: 10.1007/s00508-020-01735-5 (PMC8116226; doi:10.1007/s00508-020-01735-5)
Supplement: Supplementary file 5 — S Table 2. Changes from baseline to end of treatment in serum immunological markers. [file 508_2020_1735_MOESM5_ESM.docx]

Supplementary Table 2. Changes from baseline to end of treatment in serum immunological markers

| **Serum immunology markers** | **Mean ± SD** | | | | | | | | | |
| --- | --- | --- | --- | --- | --- | --- | --- | --- | --- | --- |
|  | **Day 0** | | | **Day 28** | | | **Delta (d28-d0)** | | | **p-value** |
| Lymphocyte gate | 95,56 | ± | 1,36 | 95,35 | ± | 1,21 | -0,22 | ± | 2,34 | 0,764 |
| Lymphocytes | 30,98 | ± | 7,99 | 28,02 | ± | 6,85 | -2,96 | ± | 8,37 | 0,268 |
| Monocytes | 5,48 | ± | 1,62 | 5,79 | ± | 1,31 | 0,31 | ± | 1,90 | 0,6 |
| Granulocytes | 61,63 | ± | 8,98 | 63,00 | ± | 6,45 | 1,37 | ± | 8,59 | 0,608 |
| CD3 | 66,19 | ± | 6,95 | 68,14 | ± | 7,29 | 1,94 | ± | 2,27 | **0,017** |
| CD19 | 11,61 | ± | 4,21 | 10,64 | ± | 3,38 | -0,97 | ± | 2,59 | 0,241 |
| CD4 | 44,66 | ± | 4,61 | 44,69 | ± | 6,11 | 0,03 | ± | 3,09 | 0,977 |
| CD8 | 27,86 | ± | 5,53 | 28,57 | ± | 6,04 | 0,72 | ± | 3,12 | 0,463 |
| CD8+/CD4+ | 0,64 | ± | 0,16 | 0,66 | ± | 0,20 | 0,02 | ± | 0,11 | 0,496 |
| CD3+/HLA-DR+ | 10,21 | ± | 6,31 | 10,08 | ± | 5,51 | -0,13 | ± | 5,49 | 0,94 |
| CD3-/HLA-DR+ | 14,30 | ± | 2,46 | 12,39 | ± | 2,25 | -1,91 | ± | 2,76 | **0,045** |
| CD3+/CD16+56+ | 5,97 | ± | 3,54 | 4,91 | ± | 3,25 | -1,06 | ± | 2,53 | 0,194 |
| CD3-/CD16+56+ | 12,59 | ± | 4,95 | 11,15 | ± | 5,09 | -1,45 | ± | 2,29 | 0,062 |
| Monocytes classical | 85,18 | ± | 4,90 | 81,73 | ± | 10,65 | -3,45 | ± | 11,06 | 0,325 |
| Monocytes intermediate | 6,00 | ± | 2,61 | 7,36 | ± | 6,73 | 1,36 | ± | 6,85 | 0,524 |
