# Supplementary material for: Open-label phase II study evaluating safety and efficacy of the non-steroidal farnesoid X receptor agonist PX-104 in non-alcoholic fatty liver disease
Source: Wien Klin Wochenschr. 2020 Sep 15;133(9):441–51. doi: 10.1007/s00508-020-01735-5 (PMC8116226; doi:10.1007/s00508-020-01735-5)
Supplement: Supplementary file 6 — Suppl 1 File. Detailed inclusion and exclusion criteria. [file 508_2020_1735_MOESM6_ESM.docx]

Supplement 1:

**Detailed inclusion and exclusion criteria:**

**Inclusion Criteria:**

- Male or females
- Age 18-70 years
- Diagnosis of NAFLD as defined as (all of the following 5 points must apply):

1. NAFLD on liver biopsy within 2 years prior to study participation OR liver steatosis on any imaging (ultrasound, MRI, CT)
2. Disturbed metabolic homeostasis based on impaired HOMA-IR > 1
3. ALT within or above the upper tertial of the ULN (ALT < 45 for males, < 35 for females U/l) and/or GGT above ULN (GGT > 55 for males, > 38 for females U/l)
4. Steatosis > 10% at CAP Fibroscan at screening
5. Steatosis > 10% equivalent to histology at 1H-MRS at baseline

- Weight > 65 kg
- BMI > 25 and < 40
- Negative blood or urine pregnancy test (for females of childbearing potential) collected at screening followed by another negative serum pregnancy test collected within 24 hours prior to the first dose of study drug.
- Female patients must be postmenopausal, surgically sterile, or if premenopausal, must be prepared to use at least two effective (≤1% failure rate) method of contraception during the course of the study and for 14 days after the end of dosing. Male patients with female partners of child bearing potential must be prepared to use at least two effective methods of contraception with all sexual partners unless they have had a prior vasectomy. Effective methods of contraception are considered to be:
  - Condom (male or female)
  - Diaphragm, with spermicide
  - Hormonal (e.g. contraceptive pill, patch, intramuscular implant or injection)
  - Intrauterine device (IUD)
  - Vasectomy (partner)
- Must be willing and able to give written informed consent and agree to comply with the study protocol.
- Sinus rhythm in 12-lead ECG

**Exclusion Criteria:**

Hepatologic (condition-specific) Exclusion Criteria:

- Evidence of excessive alcohol, drug or substance abuse (excluding marijuana use) within 1 year of first dose (>30g/day; 140g/week).
- History or other evidence of a medical condition associated with chronic liver disease other than NAFLD (e.g., hemochromatosis, viral or autoimmune hepatitis, Wilson's disease, α1-antitrypsin deficiency, alcoholic liver disease, and/or toxin exposure).
- History or other evidence of decompensated liver disease (Child-Pugh Grade B or higher), coagulopathy, hyperbilirubinemia, hepatic encephalopathy, hypoalbuminemia, ascites, hepatic encephalopathy, and bleeding from esophageal varices are conditions consistent with decompensated liver disease.
- Concomitant intake of fibrates or statins (at least 4-6 weeks before start; considering half-life of medication).

Cardiovascular Exclusion Criteria:

1. History of any structural cardiac disease requiring treatment:
   1. Any signs or symptoms of heart failure (NYHA II-IV)
   2. History of ventricular tachyarrhythmia requiring ongoing treatment
   3. History of bradyarrhythmia requiring Pacemaker treatment
   4. Relevant coronary artery disease
      1. History of myocardial infarction
      2. stable or unstable angina
2. Any clinically relevant findings in ECG (identified via 24h-Holter ECG or 12-Lead ECG) at screening:
   1. Conduction abnormalities
      1. AV-Block 2nd (Type II) or 3rd degree
      2. Pauses > 3seconds
   2. Ventricular arrhythmia
      1. Monomorphic or polymorphic ventricular ectopic beats ≥ 30 beats/ hours calculated as mean over the continuous ECG recording period.
      2. Non-sustained ventricular tachycardia (three or more consecutive ventricular beats at a rate of greater than 100 beats/min)
   3. Atrial arrhythmia
      1. Atrial ectopic beats ≥ 30 beats/ hours
      2. Re-entry supraventricular tachycardia
   4. Any type of tachycardia at rest (frequency >120/min at rest) in 12-lead ECG
   5. Sinus bradycardia <40 bpm as minimum recorded heart rate in 12-lead ECG or bradycardia <35 bpm in 24h Holter ECG
   6. Prolongation of QTc interval (QTc interval > 440 ms for male subjects or > 480 ms for female subjects) of >10% over 24 hours using the Fridericia method for QTc analysis
3. Poorly controlled hypertension, OR (2) screening or baseline blood pressure ≥ 160 mmHg for systolic OR (3) screening or baseline blood pressure ≥ 100 mmHg for diastolic blood pressure.
4. History of cerebrovascular disease:
   1. History of any stroke or transient ischemic attack

Additional Exclusion Criteria:

- Type I or II diabetes with HbA1C > 6.5% at screening and/or fasting plasma glucose > 7mmol/L (> 126 mg/dl).
- History or other evidence of a clinically relevant ophthalmologic disorder due to diabetes mellitus or hypertension or history or other evidence of severe retinopathy (e.g., cytomegalovirus, macular degeneration).
- Known sensibility to any ingredients (e.g. lactose) contained in the IMP
- All conditions that do not allow MR assessments (e.g. allergy towards contrast agent, claustrophobia, use or presence of: heart pacemaker, stents or shunts, metallic implants, ear implants,…)
- History of having received any investigational drug ≤ 3 months and/or 6 x half-life prior to the first dose of study drug or the expectation that such drugs will be used during the study. Patients enrolled in this study cannot be enrolled in another study for either research, diagnostic or treatment purposes.
- Woman with childbearing potential unless using adequate contraception (see inclusion criteria); females who are pregnant or breast feeding.
- History of severe allergic (e.g. history of anaphylactic reactions) or immunologically mediated disease [(e.g., vasculitis, cryoglobulinemia, inflammatory bowel disease, idiopathic thrombocytopenic purpura, lupus erythematosus, autoimmune hemolytic anemia, scleroderma, severe psoriasis (defined as affecting > 10% of the body, where the palm of one hand equals 1%, or if the hands and feet are affected), rheumatoid arthritis requiring more than intermittent nonsteroidal anti-inflammatory medications for management, etc.]
- Evidence of an active or suspected cancer, or a history of malignancy within the last 2 years, with the exception of patients with basal cell carcinoma that has been excised and cured.
- History of any systemic anti-neoplastic or immunomodulatory treatment (including supraphysiologic doses of steroids and radiation) 6 months prior to the first dose of study drug or the expectation that such treatment will be needed at any time during the study.
- History of bleeding disorders or anticoagulant use
- History or other evidence of chronic pulmonary disease associated with functional limitation.
- History of uncontrolled severe seizure disorder.
- Poorly controlled thyroid dysfunction.
- History of major organ transplantation with an existing functional graft.
- Any signs of acute infection or inflammation
- History or other evidence of severe illness, or any other conditions which would make the patient, in the opinion of the investigator, unsuitable for the study.
- Any herbal supplements containing silymarin, tocopherol, vitamin C, bioflavins, curcumin (at least 4-6 months before the study).
- Positive test at screening for anti-HAV IgM Ab, HBsAg, anti-HBc IgM Ab, or anti-HIV Ab.
- Subjects who have undergone surgery within the last 3 months.
- Subjects who have had a prior gastrointestinal surgery.
- Subjects who will be unavailable for the duration of the trial, who are unlikely to be compliant with the protocol, or who are felt to be unsuitable by the investigator for any other reason
- Imprisonment
