# Supplementary material for: Open-label phase II study evaluating safety and efficacy of the non-steroidal farnesoid X receptor agonist PX-104 in non-alcoholic fatty liver disease
Source: Wien Klin Wochenschr. 2020 Sep 15;133(9):441–51. doi: 10.1007/s00508-020-01735-5 (PMC8116226; doi:10.1007/s00508-020-01735-5)
Supplement: Supplementary file 7 — Suppl 2 File. Methods. [file 508_2020_1735_MOESM7_ESM.docx]

#### Supplement 2:

#### Methods:

#### Lipoprotein analysis:

Cholesterol and triglycerides were measured using enzymatic reagents (DiaSys Diagnostic Systems GmbH, Holzheim, Germany). Free fatty acids were measured enzymatically (Wako Chemicals GmbH, Neuss, Germany). Apolipoproteins were determined by immunoturbidimetry (DiaSys Diagnostic Systems GmbH, Holzheim, Germany). Lipoprotein analyses were performed on an Olympus AU640 (Olympus Diagnostika, Hamburg, Germany).

#### Clamp-like Index (CLIX)

CLIX was calculated as follows: *SC f/(mAUCglucose mAUCC-peptide) F*, where SC stands for baseline serum creatinine concentration (in milligrams per deciliter), mAUC_glucose_ and mAUC_C-peptide_ signify the AUC of plasma glucose during OGTT from 0 min to the end of the time span (in milligrams per deciliter per minute) divided by the total amount of time, respectively (1). The constant *f* is 0.85 for males and 1.0 for females. The correction factor F converts CLIX to clamp glucose infusion rates and was chosen with 6,600 as suggested (1, 2).

#### MR-spectroscopy

#### ^1^H-MRS

Hepatic fat fraction (HFF) and hepatic lipid composition were measured using single voxel PRESS sequence (TE=30 ms, TR=2000 ms, VOI: 3x3x3 cm^3^, 6 averages within acquisition time of 16 s during breathold) at 3 T (Trio Tim, Siemens Healthcare, Erlangen, Germany) with the whole body RF coil for excitation and the combination of body-matrix and spine coil, supplied by system manufacturer, for signal acquisition. Spectra were analyzed with AMARES within jMRUI (3) and fat total signal area (FTSA is equal to HFF) was assessed as the ratio of lipid signals (0.9 – 2.8 ppm) to the whole ^1^H MRS signal including that of water following appropriate T_1_ and T_2_ correction. Histology was also compared to the adapted hepatic fat fraction calculated with a non-linear function recently published by Hajek et al. (4) describing the relationship between measured hepatic fat fraction and liver histology: Histology = *-10.99+98.42*[1-exp(-0.13*ØFAT)]*, where *ØFAT is fat volume fraction calculated as 100*FTSA/(1.138 − 0.339FTSA)*. Lipid composition was assessed as lipid saturation (SI), unsaturation (UI), polyunsaturation (PUI) and monounsaturation (MUI) indices calculated as published before (5): [i.e. UI= (I_allylic_ – I_diallylic_)/(I_allylic_ + I_diallylic_ + I_methylene_ + I_methyl_)]

#### ^31^P-MRS

For ^31^P MRS, the patients were placed in the right lateral position within 7 T whole body MR system (Magnetom, Siemens Healthcare, Erlangen, Germany). Two dimensional ^31^P-MR chemical shift imaging (CSI) sequence (TE=1 ms, TR=1800 ms, 12x12 matrix interpolated to 16x16, FOV: 20 cm x 20 cm, 4 averages, acquisition time of approx. 10 min (6)) was applied on the 3 cm thick sagittal slice prescribed well in the liver parenchyma parallel to the RF surface coil (^1^H/^31^P, 10 cm in diameter, Rapid Biomedical GmbH, Rimpar, Germany). Spectroscopic field of view was carefully chosen to avoid any contamination from a superficial skeletal muscle tissue. From the ^31^P MR signal the amplitudes of phosphomonoester (PME) [phosphoethanolamine (PE) + phosphocholine (PC)], phosphodiester (PDE) [glycerophosphocholine (GPC) + glycerophosphoethanolamine (GPE)], uridine-diphosphoglucose (UDPG), nicotine adenine dinucleotide phosphate (NADPH), inorganic phosphate (Pi), phosphatidylcholine (PtdC), α- and γ-adenosine triphosphate (ATP), phosphocreatine (PCr) and total phosphorus (TP) were determined as described before (7-9). Volume of interest of 4x4 voxels with a sufficient signal-to-noise and no contamination from the skeletal muscle was chosen for further signal analysis. Individual phase and frequency shift correction was performed prior to a signal deconvolution with AMARES within jMRUI. Metabolite levels are expressed as ratio to TP or as ratio to γ-ATP signal following appropriate T_1_ correction. At the end of measurement protocol, saturation transfer experiment was performed at the sagittal slab identical to the FOV of 2D CSI sequence. The chemical exchange rate constant *k* of the Pi-to-ATP reaction and the unidirectional forward exchange flux (*F*_ATP_) and calculated as published before (10).

#### Bile acid profiles:

Using a high resolution-mass spectrometry method individual BAs (e.g. cholic acid, deoxycholic acid, chenodeoxycholic acid, lithocholic acid, ursodeoxycholic acid) were determined as unconjugated acids, taurine and glycine conjugates. Free acids and corresponding conjugates were measured by full scan-high resolution-mass spectrometry, using Orbi-Trap technology, within one high-performance liquid chromatography run. High performance liquid chromatography was performed on a reversed-phase (C_18_) column using a methanol/water gradient for chromatographic solution of isobaric BAs. Quantitation was performed by the use of deuterated internal standards and correlation of peak area ratios in linear regression (11).

#### FACS analysis:

Samples were incubated with reagents containing antibodies labeled with FITC or PE. 100µl of whole blood were stained for 30 minutes at room temperature in the dark using six different reagents. A: CD45CD14; B: IgG1/IgG2a; C: CD3/CD19; D:CD4/CD8; E: CD3/HLA-DR and F: CD3/CD16+CD56. Subsequently red cells were lysed for 10 minutes (BD FACS lysing solution, Becton Dickinson) and washed afterwards three times with PBS. Washed cells were immediately analyzed on a LSRFortessa SORP (Becton Dickinson). At least 10,000 FSC/SSC gated lymphocyte cells were counted. The percentages of lymphocyte subpopulation were calculated using the software package FACS Diva 6.1 (Becton Dickinson). Ref?

#### Microbiota analysis:

Bacterial DNA from stool samples was extracted with the PowerLyzer PowerSoil DNA Isolation Kit (MO BIO Laboratories Inc, CA, USA) according to the manufacturer’s instructions. DNA concentration was measured by Picogreen fluorescence. The variable V1–V2 region of the bacterial 16S rRNA gene was amplified with PCR from 50ng DNA using oligonucleotide primers 16s_515_S3_fwd: GATTGCCAGCAGCCGCGGTAA and 16s_806_S2_rev: GGACTACCAGGGTATCTAAT. This 16S rDNA region was chosen since it gives robust taxonomic classification and has been shown to be suitable for community clustering. Bacterial 16S rRNA was amplified with the Mastermix 16s Complete PCR Kit (Molzym, Bremen, Germany). The first PCR reaction product was subjected to a second round of PCR with primers fusing the 16s primer sequence to the A and P adapters necessary for Ion Torrent sequencing while additionally including a molecular barcode sequence to allow multiplexing of up to 96 samples simultaneously. PCR products were subjected to agarose gel electrophoresis and the band of the expected length (330nt) was excised from the gel and purified using the QiaQick (Qiagen, Hilden, Germany) gel extraction system. DNA concentration of the final PCR product was measured by Picogreen fluorescence. Amplicons from up to 60 samples were pooled equimolarly and subjected to emulsion PCR using the Ion Torrent One Touch 2.0 Kit according to manufacturers protocols. After emulsion PCR the beads were purified on Ion ES station and loaded onto Ion Torrent 318 chips for sequencing. Sequencing reactions were performed on Ion Torrent PGM using the Ion 400BP Sequencing Kit running for 1082 flows (all reagents from Thermo Fisher Scientific, MA, USA). Sequences were split by barcode and transferred to the Torrent suite server. Unmapped bam files were used as input for bioinformatics.

All sequences were initially trimmed by a sliding window quality filter with a width of 20nt and a cutoff of Q20. Reads shorter than 100 nucleotides and reads mapping to the human genome were removed using DeconSeq (12). The resulting reads were subjected to error correction using the Acacia tool (13) leading to error correction of 10-20% of reads. Subsequently PCR chimeras were removed by usearch algorithm in de-novo and reference based settings (14). The final sequence files were then analyzed by QIIME 1.8 workflow scripts (15). OTU search was performed using the parallel_pick_open_reference_otus workflow script and the Greengenes 13_8 reference database. OTUs were visualized as OTU tables, bar charts and PCOA plots using the Qiime core microbiome script. Additionally groupings supplied in the mapping file were tested for statistical significance using the QIIME implementation of the Adonis test and significance of individual bacterial strains was determined by Kruskal-Wallis test.

#### Sucrose-lactulose-mannitol test:

Different sugars (10g lactulose, 20g sucrose and 5g mannitol dissolved in 100 ml H2O) were administered orally (1150 mosmol/L). Urinary excretion (self-collected at home) before oral sugar administration (baseline) and after 5 hours was assessed by high-performance liquid chromatography, leading to a permeability index. Cut-off is > 0,03 (lactulose/mannitol index) and > 43 mg sucrose excretion representing elevated intestinal permeability (as in untreated celiac disease). Additionally, 2g Sucralose were administered orally together with the other sugars. Urinary excretion from 5 hours to 24 hours was collected in a separate container, resulting in a colonic permeability index measuring total excreted sucralose from 0 to 24 hours.

1. Anderwald C, Anderwald-Stadler M, Promintzer M, Prager G, Mandl M, Nowotny P, Bischof MG, et al. The Clamp-Like Index: a novel and highly sensitive insulin sensitivity index to calculate hyperinsulinemic clamp glucose infusion rates from oral glucose tolerance tests in nondiabetic subjects. Diabetes care 2007;30:2374-2380.

2. Mari A, Pacini G, Murphy E, Ludvik B, Nolan JJ. A model-based method for assessing insulin sensitivity from the oral glucose tolerance test. Diabetes Care 2001;24:539-548.

3. Vanhamme L, van den Boogaart A, Van Huffel S. Improved method for accurate and efficient quantification of MRS data with use of prior knowledge. J Magn Reson 1997;129:35-43.

4. Hájek M, Dezortová M, Wagnerová D, Skoch A, Voska L, Hejlová I, Trunečka P. MR spectroscopy as a tool for in vivo determination of steatosis in liver transplant recipients. MAGMA 2011;24:297-304.

5. Johnson NA, Walton DW, Sachinwalla T, Thompson CH, Smith K, Ruell PA, Stannard SR, et al. Noninvasive assessment of hepatic lipid composition: Advancing understanding and management of fatty liver disorders. Hepatology 2008;47:1513-1523.

6. Chmelik M, Kukurova IJ, Gruber S, Krssak M, Valkovic L, Trattnig S, Bogner W. Fully adiabatic 31P 2D-CSI with reduced chemical shift displacement error at 7 T--GOIA-1D-ISIS/2D-CSI. Magn Reson Med 2013;69:1233-1244.

7. Chmelik M, Považan M, Krssak M, Gruber S, Tkačov M, Trattnig S, Bogner W. In vivo (31)P magnetic resonance spectroscopy of the human liver at 7 T: an initial experience. NMR Biomed 2014;27:478-485.

8. Cortez-Pinto H, Chatham J, Chacko VP, Arnold C, Rashid A, Diehl AM. Alterations in liver ATP homeostasis in human nonalcoholic steatohepatitis: a pilot study. JAMA 1999;282:1659-1664.

9. Chmelík M, Schmid AI, Gruber S, Szendroedi J, Krssák M, Trattnig S, Moser E, et al. Three-dimensional high-resolution magnetic resonance spectroscopic imaging for absolute quantification of 31P metabolites in human liver. Magn Reson Med 2008;60:796-802.

10. Valkovic L, Gajdosik M, Traussnigg S, Wolf P, Chmelik M, Kienbacher C, Bogner W, et al. Application of localized (3)(1)P MRS saturation transfer at 7 T for measurement of ATP metabolism in the liver: reproducibility and initial clinical application in patients with non-alcoholic fatty liver disease. Eur Radiol 2014;24:1602-1609.

11. Amplatz B, Zohrer E, Haas C, Schaffer M, Stojakovic T, Jahnel J, Fauler G. Bile acid preparation and comprehensive analysis by high performance liquid chromatography-high-resolution mass spectrometry. Clin Chim Acta 2016;464:85-92.

12. Schmieder R, Edwards R. Fast identification and removal of sequence contamination from genomic and metagenomic datasets. PLoS One 2011;6:e17288.

13. Bragg L, Stone G, Imelfort M, Hugenholtz P, Tyson GW. Fast, accurate error-correction of amplicon pyrosequences using Acacia. Nat Methods 2012;9:425-426.

14. Edgar RC. Search and clustering orders of magnitude faster than BLAST. Bioinformatics 2010;26:2460-2461.

15. Caporaso JG, Kuczynski J, Stombaugh J, Bittinger K, Bushman FD, Costello EK, Fierer N, et al. QIIME allows analysis of high-throughput community sequencing data. Nat Methods 2010;7:335-336.
